# Supplementary material for: Augmented Concentration of Isopentyl-Deoxynyboquinone in Tumors Selectively Kills NAD(P)H Quinone Oxidoreductase 1-Positive Cancer Cells through Programmed Necrotic and Apoptotic Mechanisms
Source: Cancers (Basel). 2023 Dec 14;15(24):5844. doi: 10.3390/cancers15245844 (PMC10741405; doi:10.3390/cancers15245844)
Supplement: Supplementary file 1 [file cancers-15-05844-s001.zip › cancers-2689142-supplementary/cancers-2689142-supplementary.pdf]

SUPPLEMENTARY MATERIALS for

Augmented Concentration of Isopentyl-Deoxynyboquinone in Tumors Selectively Kills NAD(P)H Quinone Oxidoreductase 1-positive Cancer Cells through Programmed Necrotic and Apoptotic Mechanisms

Jiangwei Wang, Xiaolin Su, Lingxiang Jiang, Matthew W. Boudreau, Lindsay E. Chatkewitz, Jessica A. Kilgore, Kashif Rafiq Zahid, Noelle S. Williams, Yaomin Chen, Shaohui Liu, Paul J. Hergenrother and Xiumei Huang

This file includes the following:

Figure S1. Structure of the drugs, and NQO1 protein levels and enzymatic activities in various cancer cells.

Figure S2. IP-DNQ induces PAR formation.

Figure S3. Absence of NQO1 expression impedes cell proliferation, and compensatory mechanisms result in similar rates of tumor growth in vivo.

Figure S4. Measurement of plasma ALT and AST enzyme activities for liver function.

Supplementary Table 1

Supplemental Experimental Procedures

Supplementary Figure S1

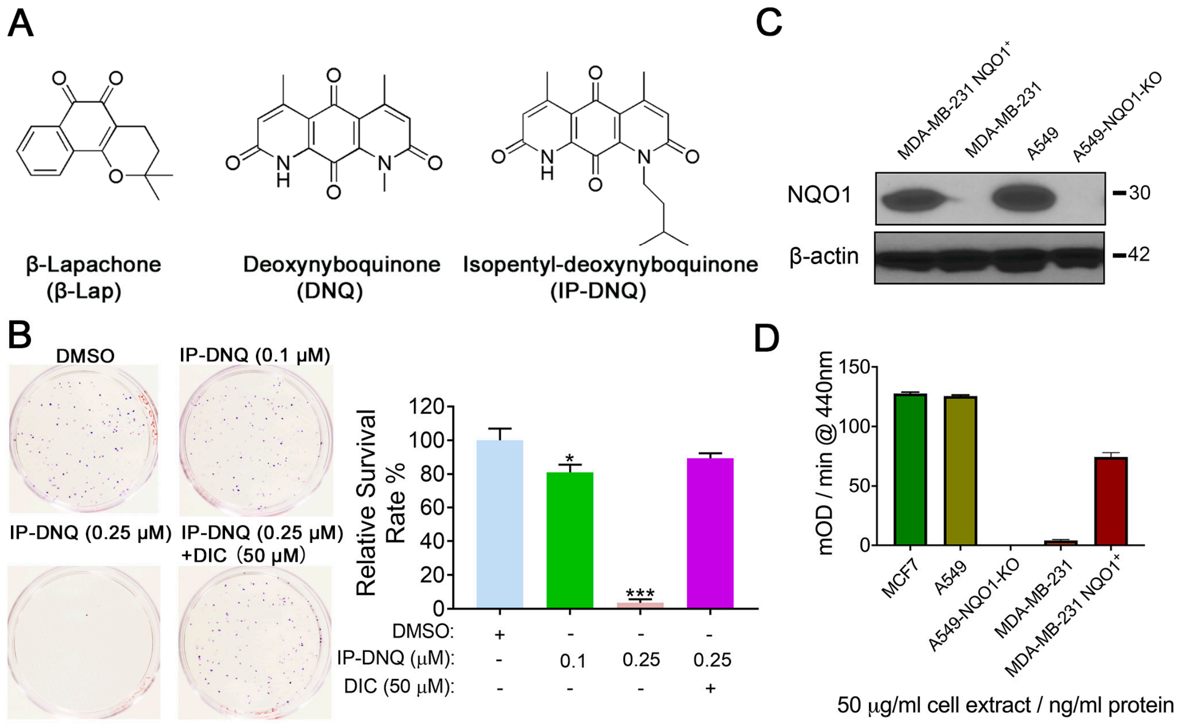

Figure S1. Structure of the drugs, and NQO1 protein levels and enzymatic activities in various cancer cells. **(A)** Structures of β-lapachone (β-lap), deoxynyboquinone (DNQ), and isopentyl-deoxynyboquinone (IP-DNQ). **(B)** A549 cells were treated with DMSO or IP-DNQ (0.1 or 0.25 µM) for 2 h, ± DIC (50 µM). Drugs were removed, and cell viability was determined with a colony formation assay 7 days later. **(C)** NQO1 expression in breast cancer MDA-MB-231 ± NQO1$^+$ cells, NSCLC A549, and A549-NQO1 cells. **(D)** NQO1 enzyme activity in breast cancer MCF-7, MDA-MB-231-NQO1$^+$, and MDA-MB-231 cells, and NSCLC A549 and A549-NQO1-KO cells. Results (mean ± SD) were derived from three independent experiments. ***$p < 0.001$, **$p < 0.01$, *$p < 0.05$ (t tests).

Supplementary Figure S2

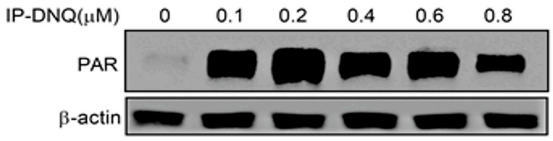

Figure S2. IP-DNQ induces PAR formation.
A549 cells were exposed to DMSO and various dosages of IP-DNQ (0.1 - 0.8 μM) for 5 min. Samples were assessed for PAR formation and β-actin (loading control).

Supplementary Figure S3

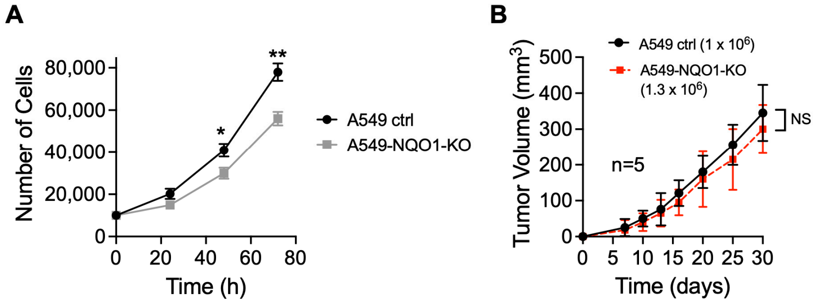

Figure S3. Absence of NQO1 expression impedes cell proliferation, and compensatory mechanisms result in similar rates of tumor growth in vivo.
A. Proliferation rates of A549 ctrl and A549-NQO1-KO cells were measured at 0, 24, 48, and 72 hours using the CyQuant cell proliferation Kit from Life Technologies. **B.** Female NSG mice (n = 5/group) were subcutaneously inoculated with 1 x 10^6 A549 ctrl or 1.3 x 10^6 A549-NQO1-KO cells. Average tumor volumes were quantified on the days indicated in Fig S3B. For panel A, results (mean ± SD) were derived from three independent experiments. $^{**}p < 0.01$, $^{*}p < 0.05$ (*t*-tests); NS, no significance.

Supplementary Figure S4

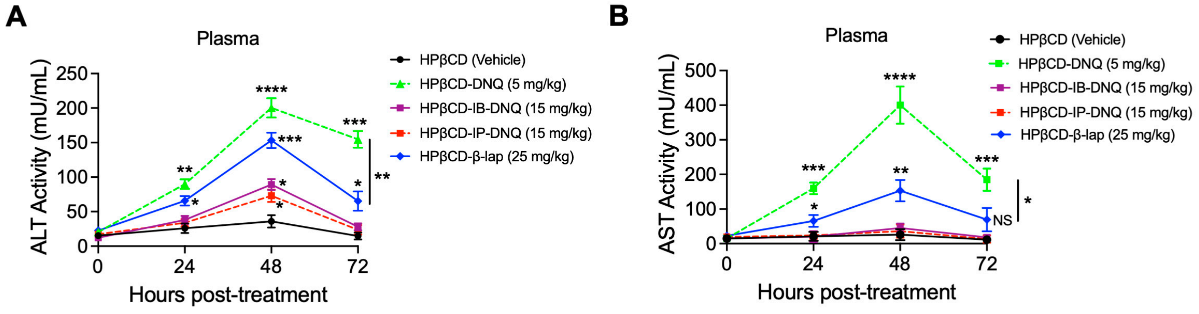

Figure S4. Measurement of plasma ALT and AST enzyme activities for liver function.
Plasma ALT (A) and AST (B) activities (mU/mL) in female NSG mice (n=3/group) treated with vehicle (HPβCD), HPβCD-DNQ (5 mg/kg), HPβCD-IB-DNQ (15 mg/kg), HPβCD-IP-DNQ (15 mg/kg), or HPβCD-β-lap (25 mg/kg) via tail vein i.v. injection for one dose; they were sacrificed at the given time points. Mice were sacrificed at various times (from 0 to 72 h) post-drug treatment. Whole blood was collected. Blood samples were separated by centrifugation for 10 minutes at 9600 x g, and the plasma supernatant was used for ALT and AST enzyme activity assays. Results (mean ± SD) were from three independent experiments. ****$p < 0.0001$, ***$p < 0.001$, **$p < 0.01$, *$p < 0.05$ (t-tests); NS indicates no significance.

Supplementary Table S1.

Symptoms after dosing	β-Lap	DNQ	IB-DNQ	IP-DNQ
MTD	25 mg/kg	5 mg/kg	15 mg/kg	15 mg/kg
Tachypnea and jumping	Moderate	Severe	Less	Less
Hunched down	Moderate	Severe	Less	Less
Stand up again	About 1-2 h	> 3-5 h	< 0.5 h	< 0.5 h
Hypoactivity days	~ 1 day	3-4 days	~ 0.5 day	~ 0.5 day
Ruffled fur	Yes	Yes	No	No
Tachypnea	2	3	1	1
Jumping	2	4	0	0
Hunched backs	3	4	1	1

Tachypnea (Rapid Breathing) Scoring:

0: Normal breathing;
1: Mildly increased respiratory rate;
2: Moderately increased respiratory rate with occasional abdominal breathing;
3: Severely increased respiratory rate with constant abdominal breathing and occasional pauses;
4: Labored breathing with cyanosis (blue or purple coloration due to lack of oxygen).

Jumping Behavior Scoring:

0: No jumping observed;
1: Occasional jumps, no apparent reason;
2: Frequent jumping, appears agitated;
3: Constant jumping, inability to rest;
4: Jumping with vocalizations or other signs of distress.

Hunched Posture Scoring:

0: Normal posture, active;
1: Mildly hunched posture, active;
2: Moderately hunched posture, reduced activity;
3: Severely hunched posture, minimal activity;
4: Severely hunched posture, lethargic or immobile.

Supplemental Experimental Procedures

1. Cell proliferation assays

Cell proliferation rates were assessed using the CyQUANT Cell Proliferation Assay Kit (Life Technologies) in strict adherence to the manufacturer's instructions. Initially, the standard curves were established by resuspending cell pellets, each containing 1 x 10^6 cells from various cell lines, in 1 mL of CyQUANT GR/cell-lysis buffer followed by a brief vortex. In a 96-well plate, a dilution series was created for each cell line, ranging from 50 to 50,000 cells in 200 µL volumes, alongside a 200 µL cell-free control well. The plate was incubated for 5 minutes and shielded from light at room temperature. Fluorescence was recorded using a Synergy-H1 Hybrid Reader (BioTek) at an excitation of 480 nm and an emission of 520 nm. To quantify the proliferation, 5,000 cells were seeded per well in six wells per plate, each with a 200 µL volume. Several plates, all starting from the same cell concentration, were prepared and grown at 37°C with 10% CO2 until the appropriate collection times. The plates were collected at 0, 24, 48, and 72 hours post-seeding by inverting and blotting them to discard the media. Afterward, the plates were frozen at -80°C until the collection was complete. For the analysis, the plates were thawed to room temperature, treated with 200 µL of CyQUANT GR/cell-lysis buffer each, and incubated for 5 minutes in darkness at room temperature.

2. Pharmacokinetic Analyses of HPβCD-IP-DNQ and HPβCD-IB-DNQ in Mice Bearing A549 NSCLC Xenografts

Pharmacokinetic studies were performed in NOD/SCID mice bearing orthotopic A549 lung cancer cells. The mice (n=3/group) were injected with HPβCD- IP-DNQ (12 mg/kg) or HPβCD-IB-DNQ (12 mg/kg) via the tail vein (i.v.). The animals were sacrificed at different time points (from 5 to 120 min) after the drug treatments. The whole blood and tumor tissues were harvested at the same time. The blood samples were separated by centrifugation for 10 min at 9600 x g, and the plasma supernatant was saved. The tumor tissues were homogenized in PBS. For the standards, blank commercial plasma (Bioreclamation, Westbury, NY) or an untreated tumor tissue homogenate was spiked with varying concentrations of the compound. The standards and samples were mixed with a 2X volume of 100% acetonitrile containing 0.15% formic acid, vortexed, and then spun for 5 min at 16,100 x g. The supernatant was removed and spun again, and the resulting second supernatant was put into an HPLC vial with an insert and then analyzed by LC-MS/MS

using an AB Sciex 3200 QTRAP® coupled to a Shimadzu Prominence LC. IP-DNQ and IB-DNQ were detected with the mass spectrometer in MRM (multiple reaction monitoring) mode by following the precursor to fragment ion transition as follows: 243.1 → 187.2 and 324.1 → 293.2, respectively. An Agilent Zorbax XDB-C18 column (50 x 4.6 mm, 5 micron packing) was used for chromatography with the following conditions: Buffer A: dH_2O + 0.1% formic acid; Buffer B: MeOH + 0.1% formic acid, 1.5 mL/min flow rate, 0-1.5min 3%B, 1.5-2.5 min gradient to 100% B, 2.5-3.5 min 100% B, 3.5-3.6 min gradient to 3% B, 3.6-4.5 min 3% B. In general, the back-calculations of the standard curve and quality control samples were accurate to within 20% for 70% of these samples at concentrations ranging from 5 ng/ml to 10,000 ng/ml. The pharmacokinetic parameters from 0 to 2 h for IP-DNQ/IB-DNQ were calculated using the non-compartmental analysis tool of Phoenix WinNonlin (Certara Corporation, Princeton, NJ). An unpaired t-test (GraphPad QuickCalcs, San Diego, CA) was used to test for significant differences in the IP-DNQ and IB-DNQ concentrations in the different treatment groups.

3. Measuring ALT and AST Enzyme Activities

The liver enzyme activities, ALT (Alanine Transaminase) and AST (Aspartate Aminotransferase), were measured in plasma treated with EDTA utilizing the Alanine Transaminase Activity Assay Kit (ab105134) and the Aspartate Aminotransferase Activity Assay Kit (ab138878), respectively, both sourced from Abcam. The procedures were conducted in accordance with the guidelines provided by the manufacturer. All samples were processed concurrently and assessed in triplicate wells for each mouse sample, and the average of these technical replicates was employed for further analysis.
