# Supplementary figures and images for: Augmented Concentration of Isopentyl-Deoxynyboquinone in Tumors Selectively Kills NAD(P)H Quinone Oxidoreductase 1-Positive Cancer Cells through Programmed Necrotic and Apoptotic Mechanisms
Source: Cancers (Basel). 2023 Dec 14;15(24):5844. doi: 10.3390/cancers15245844 (PMC10741405; doi:10.3390/cancers15245844)

Figure 2D

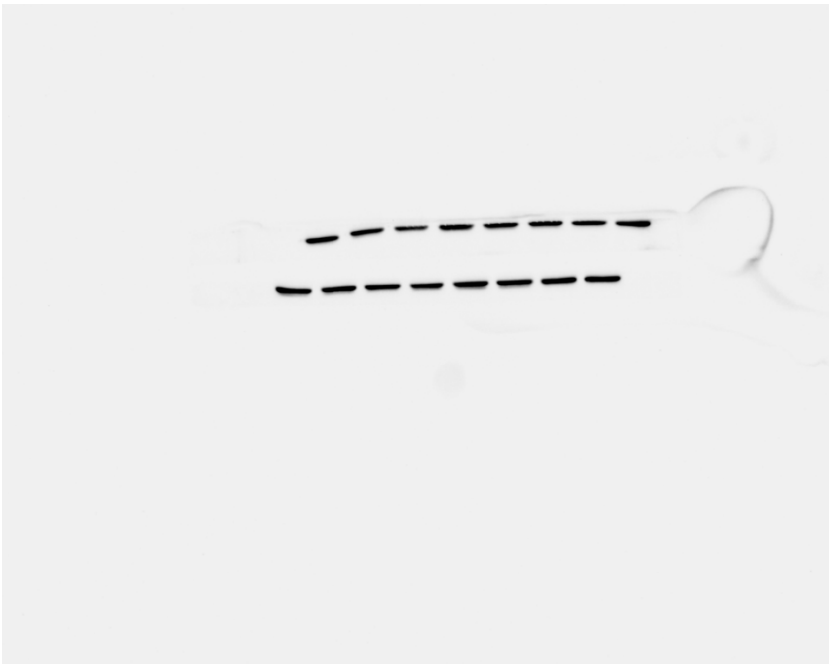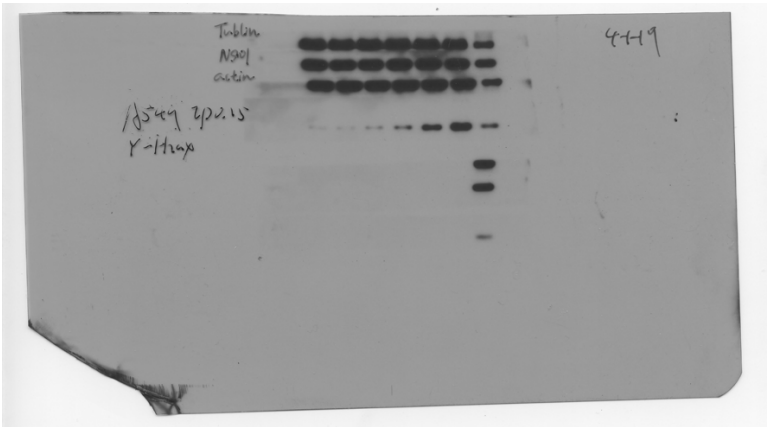

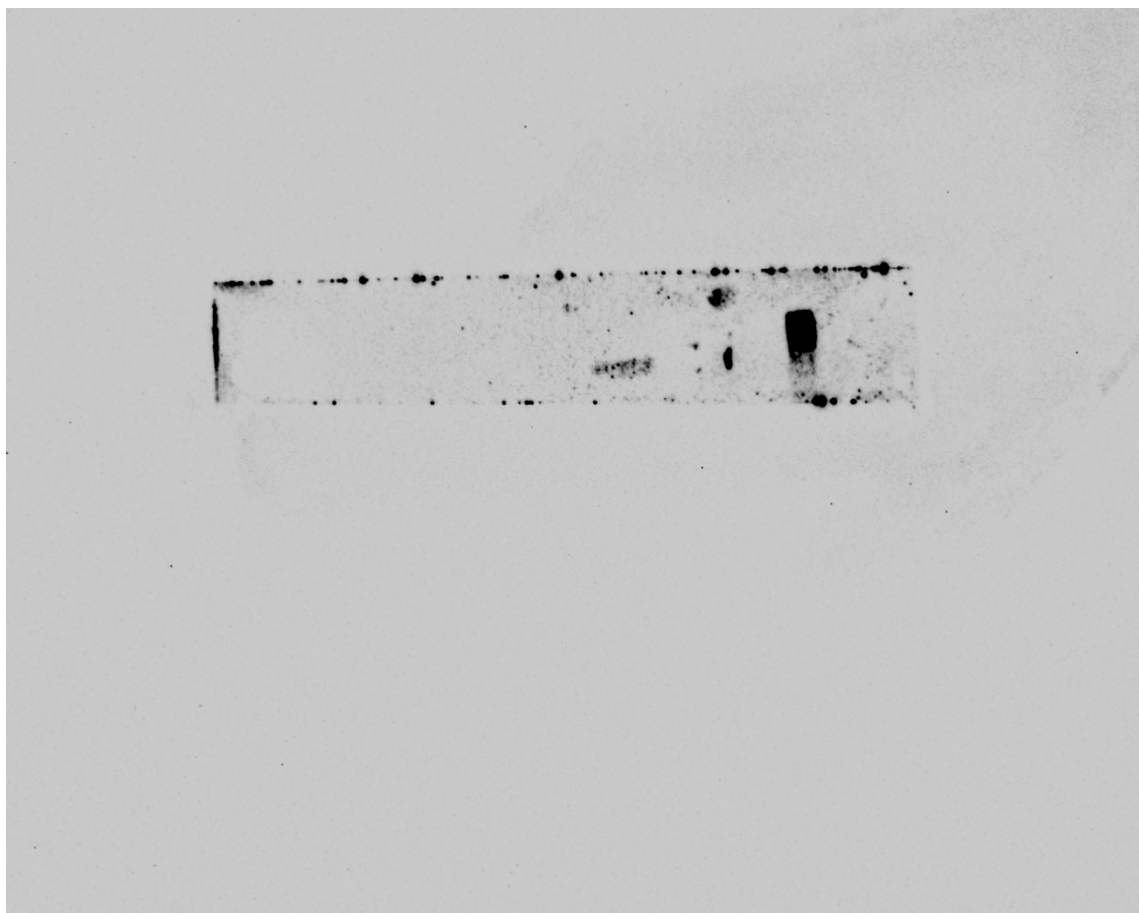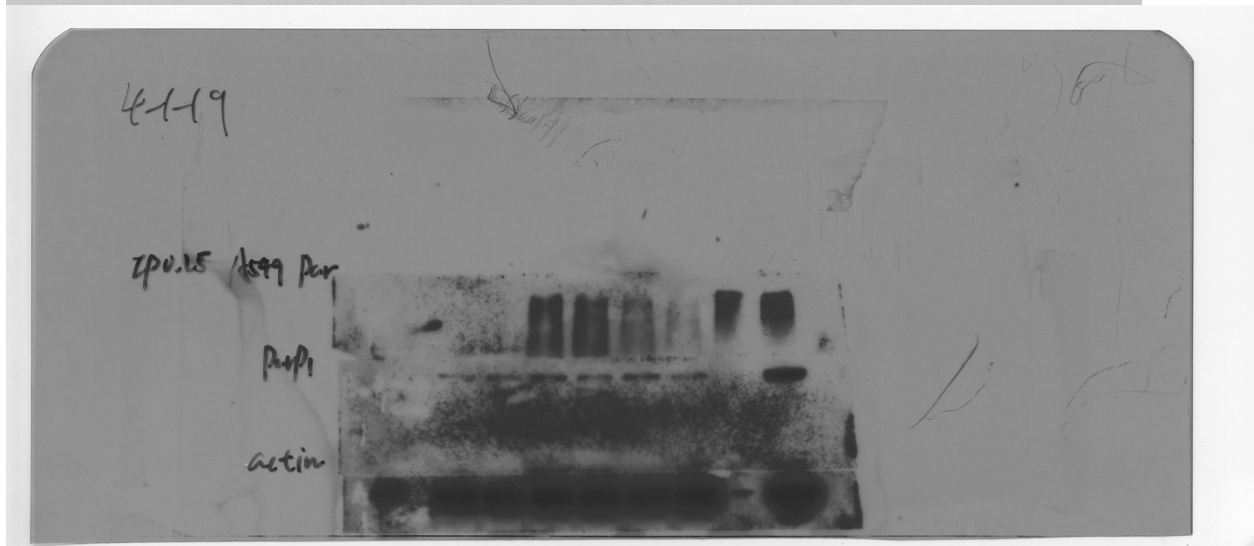

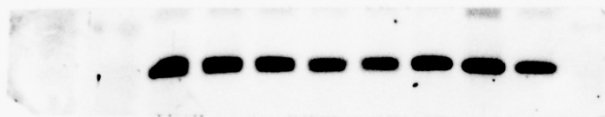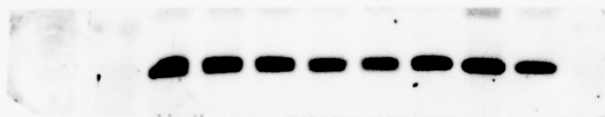

Figure 2E

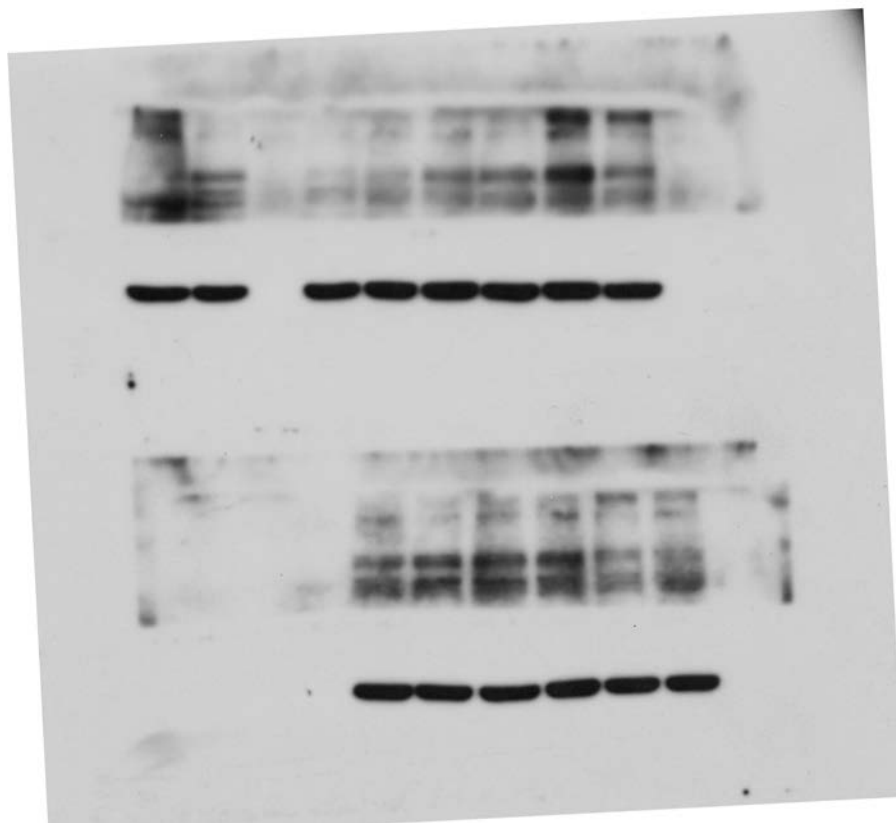

Figure 2F.

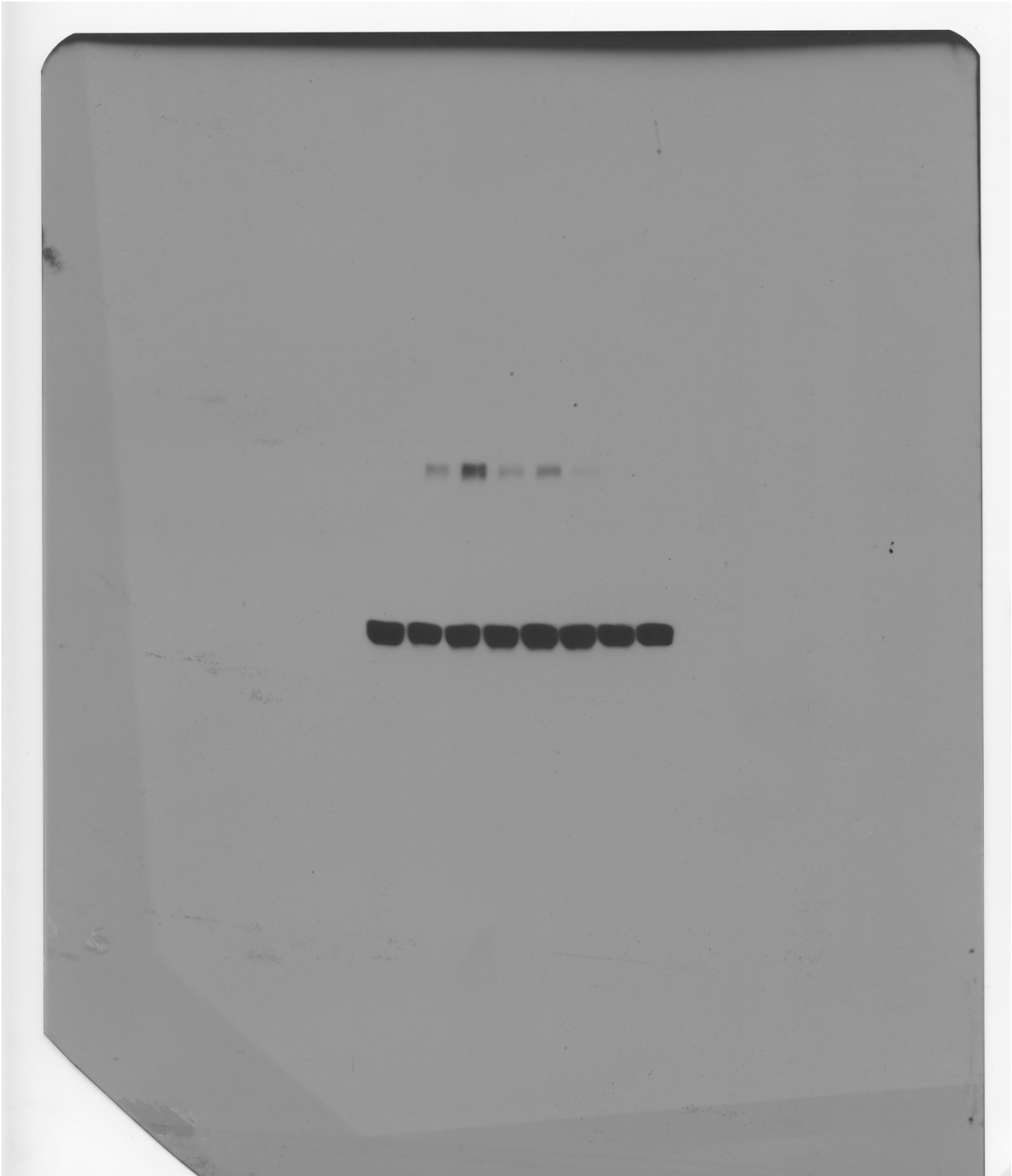

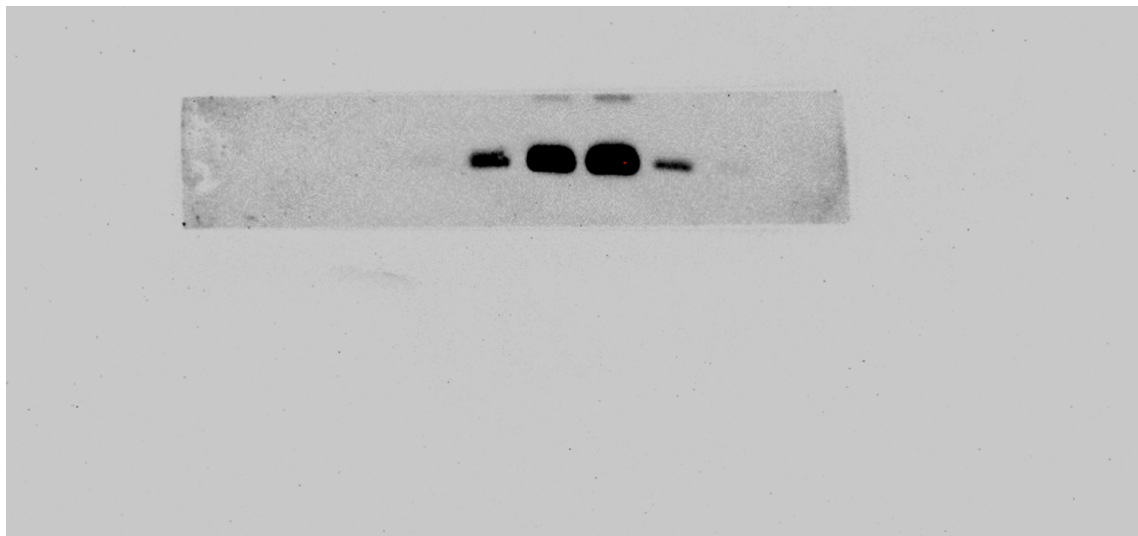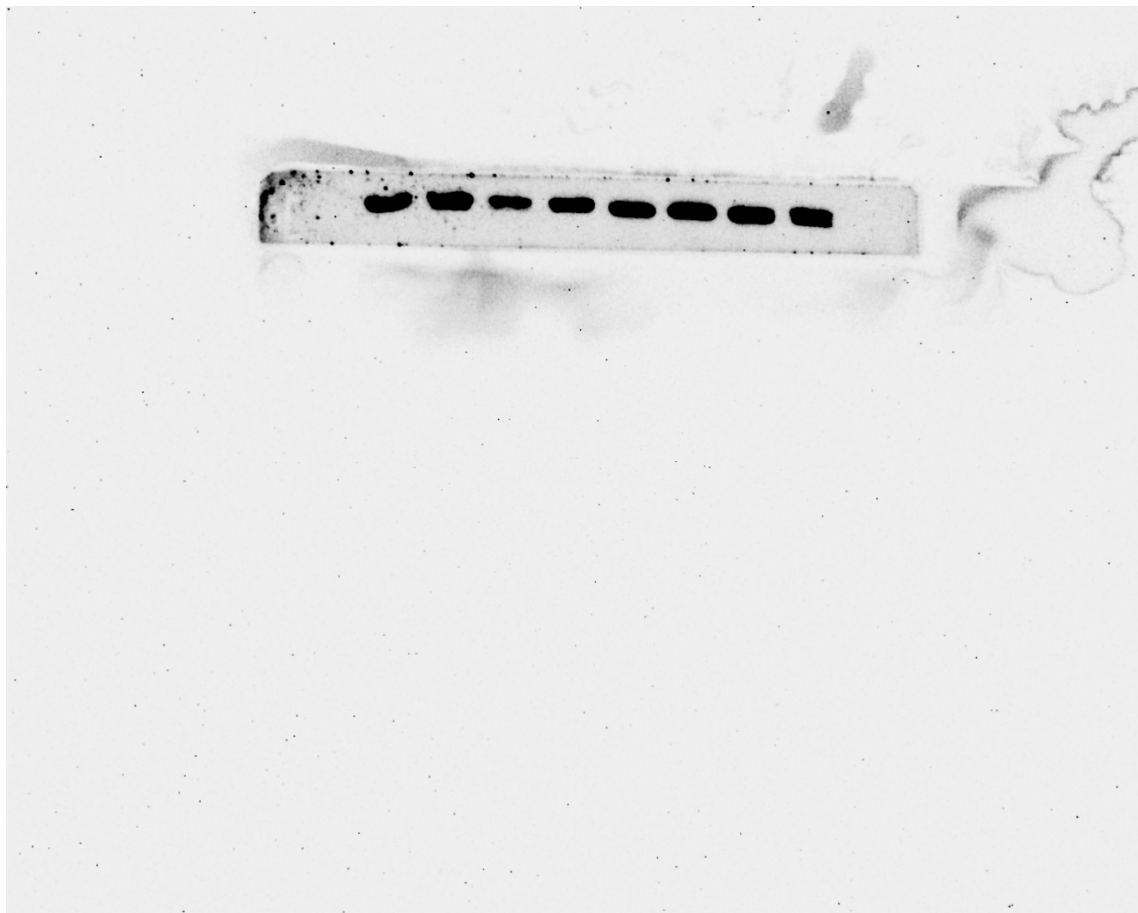

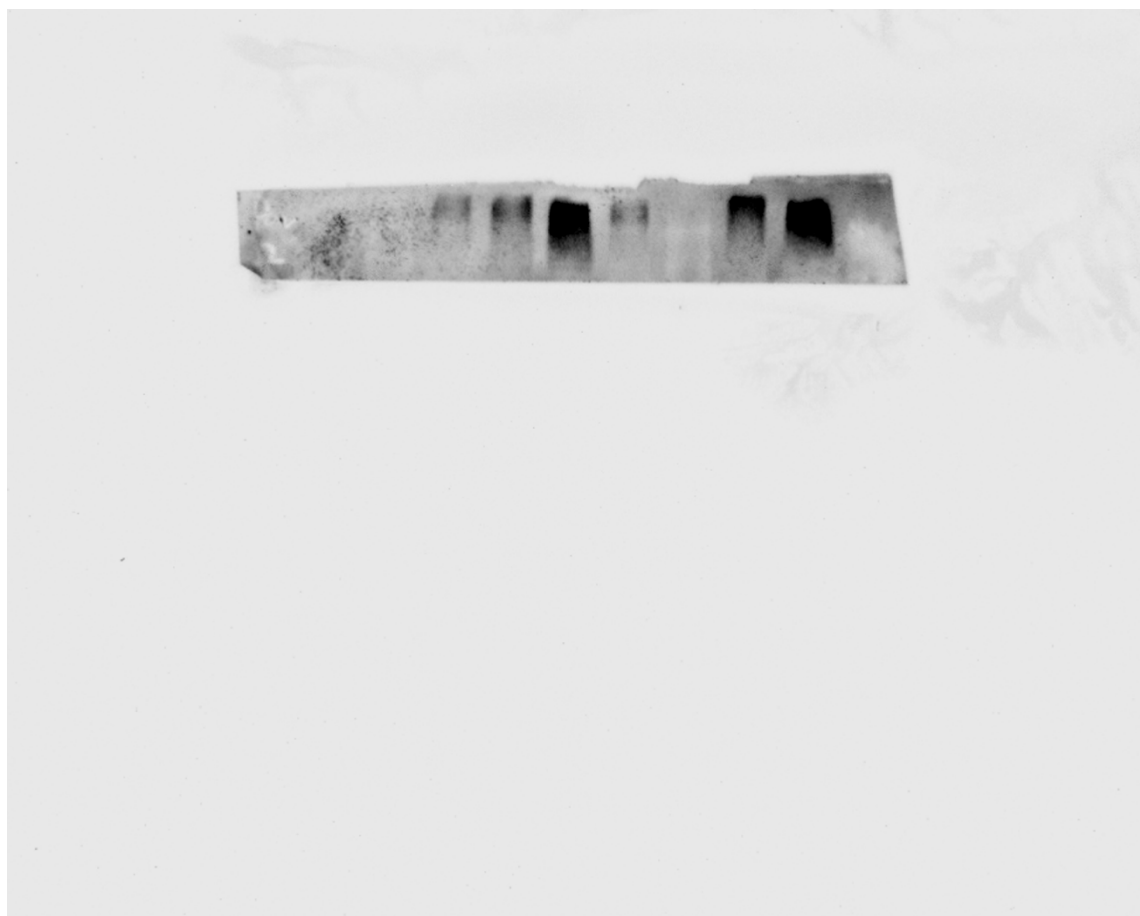

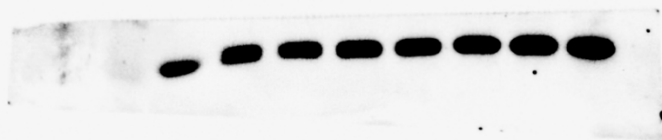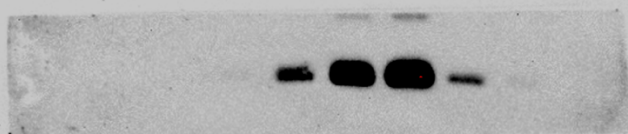

Figure 4B

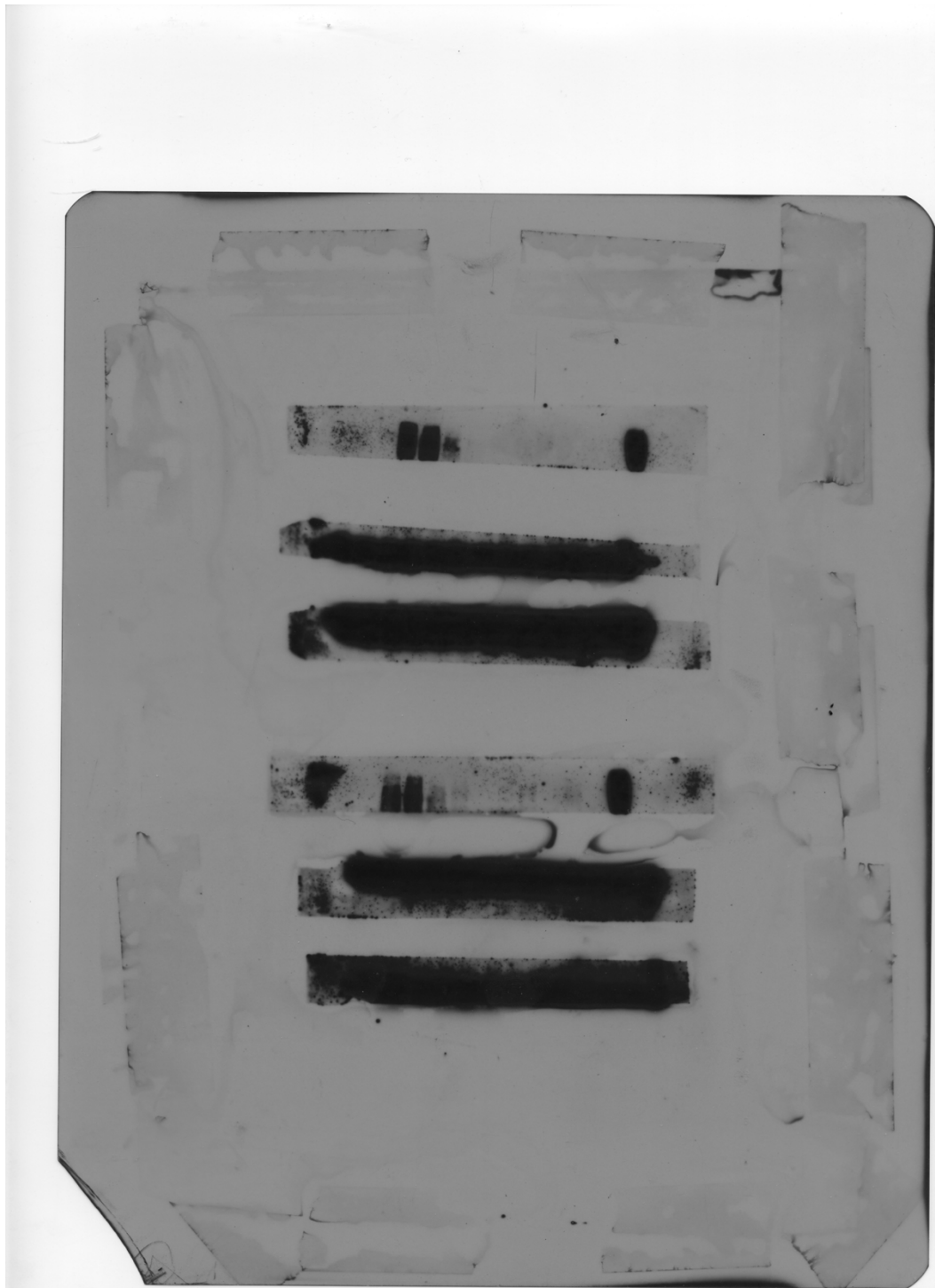

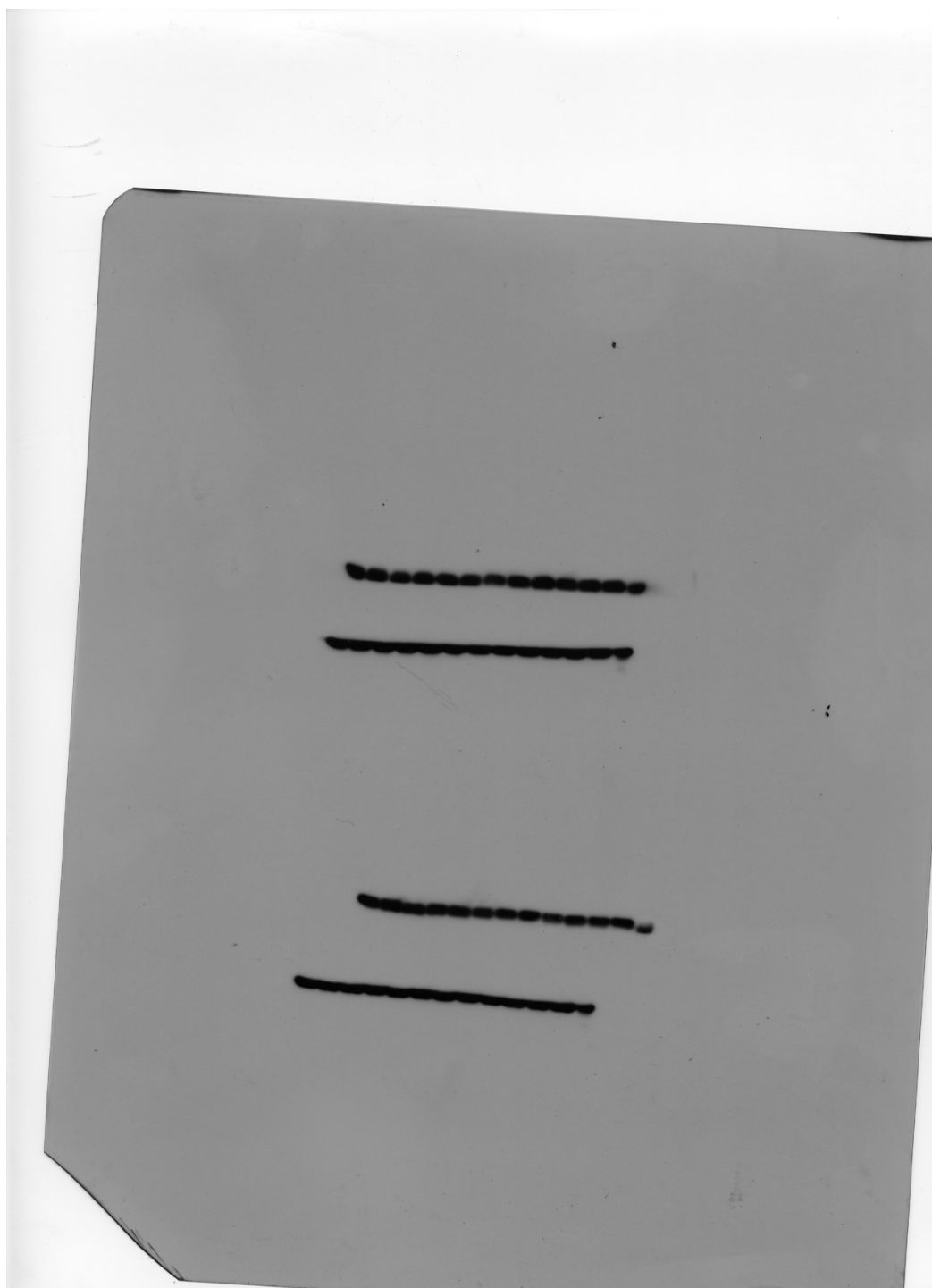

Figure 5B

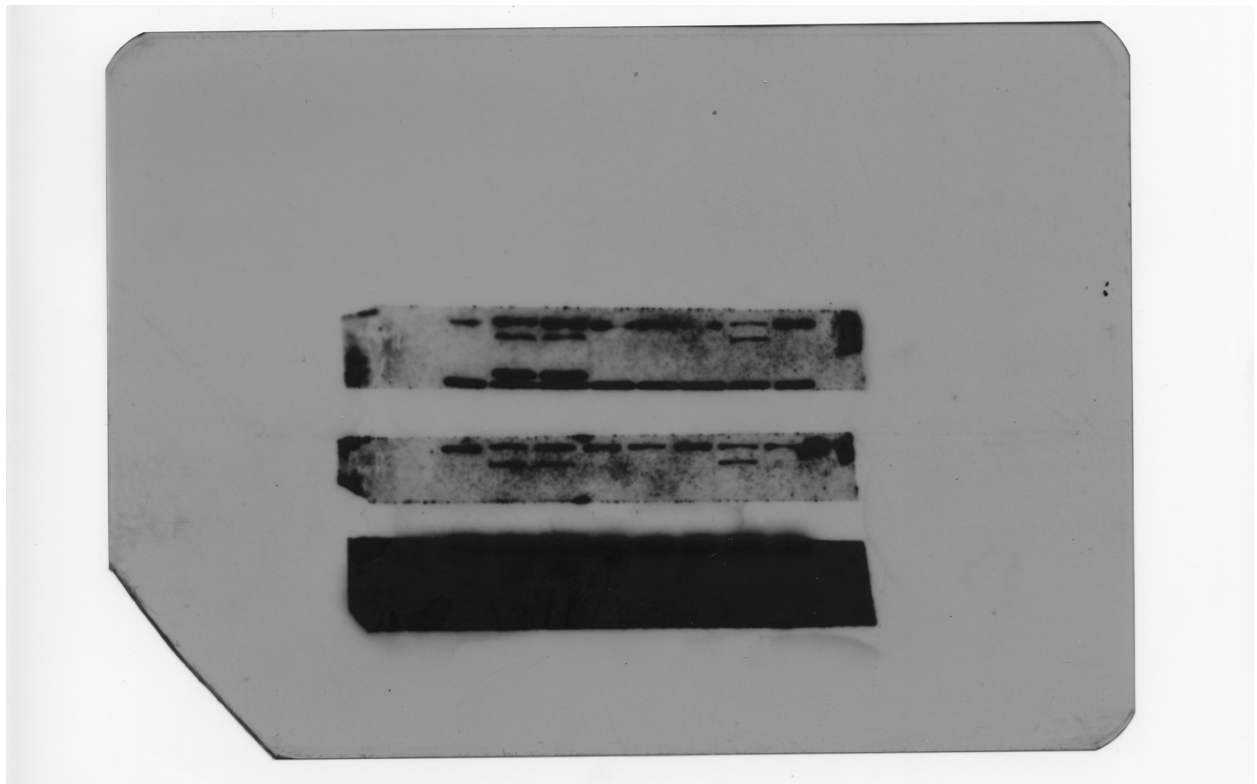

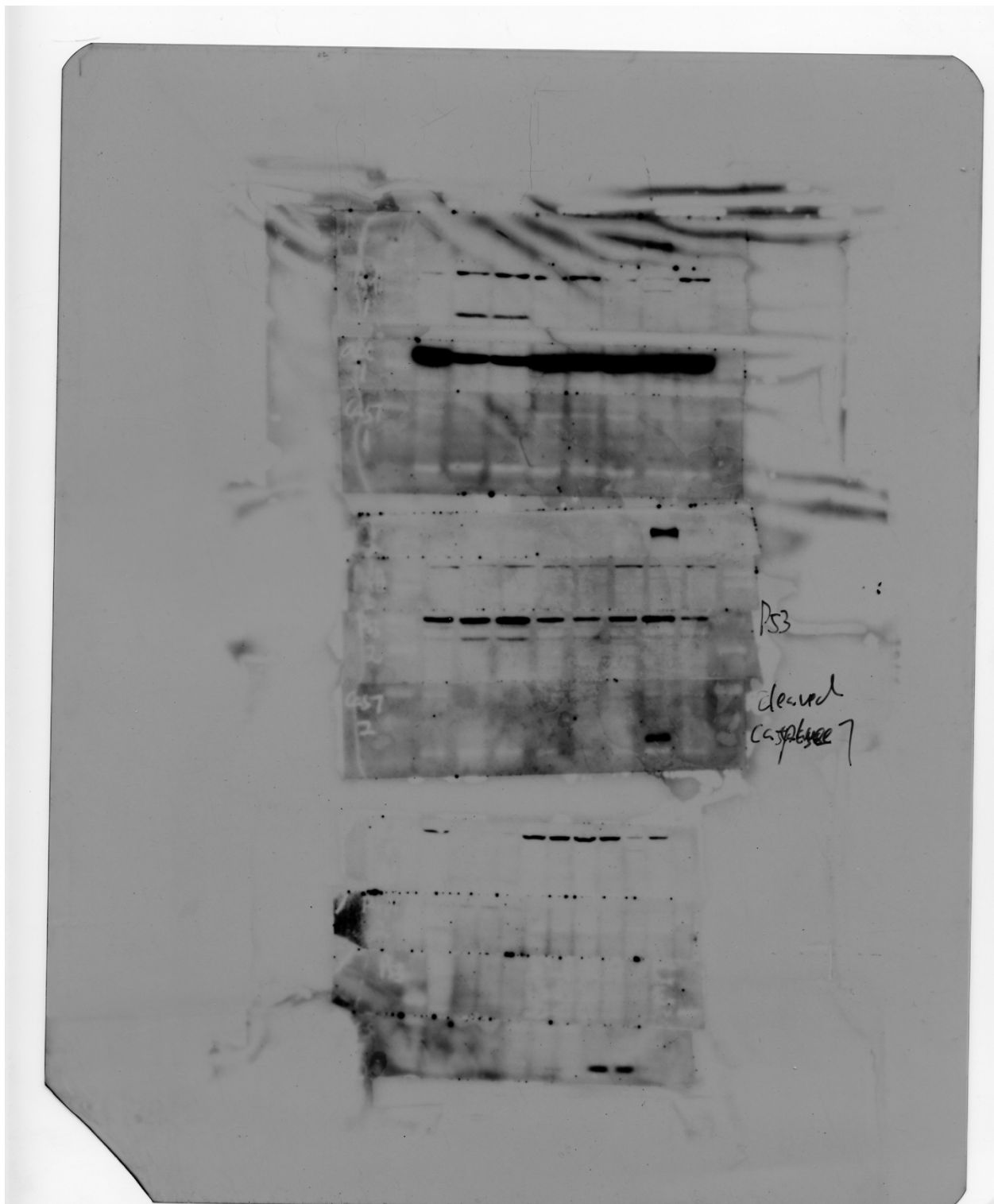

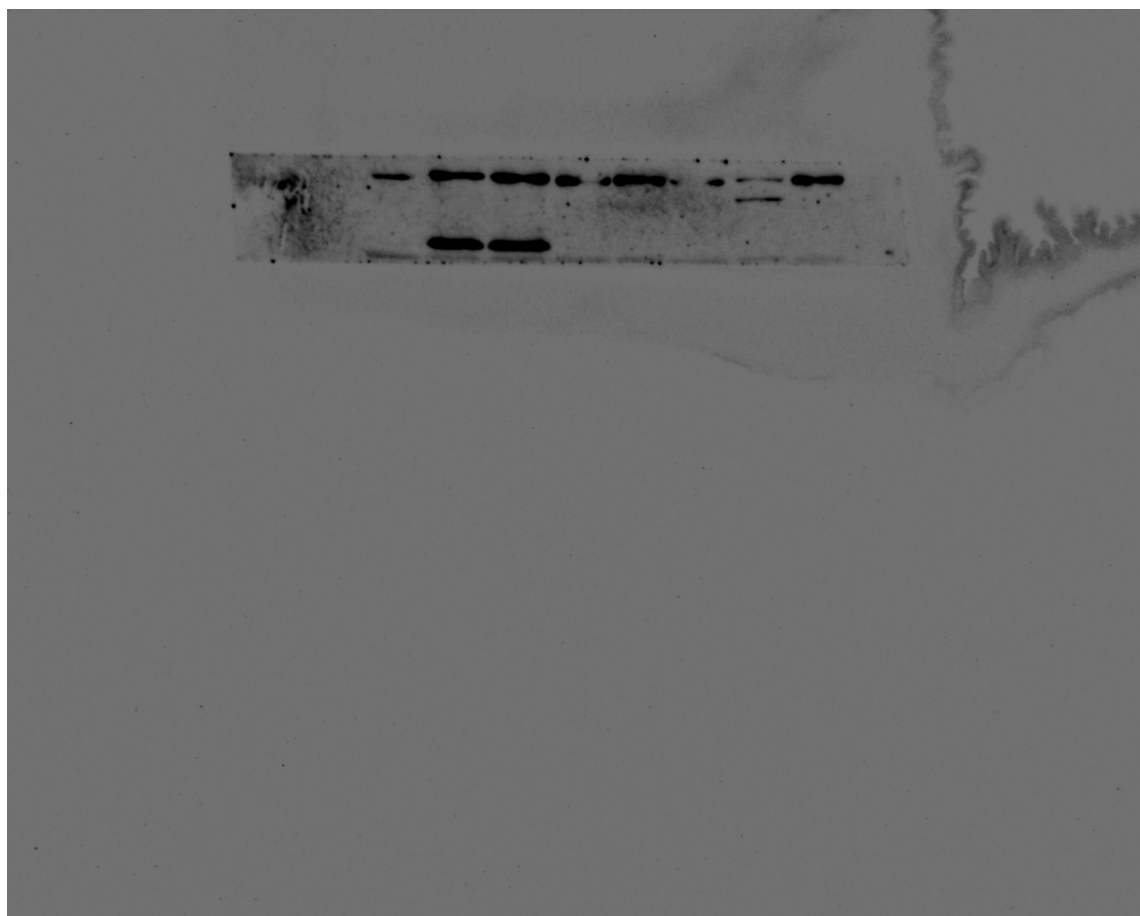

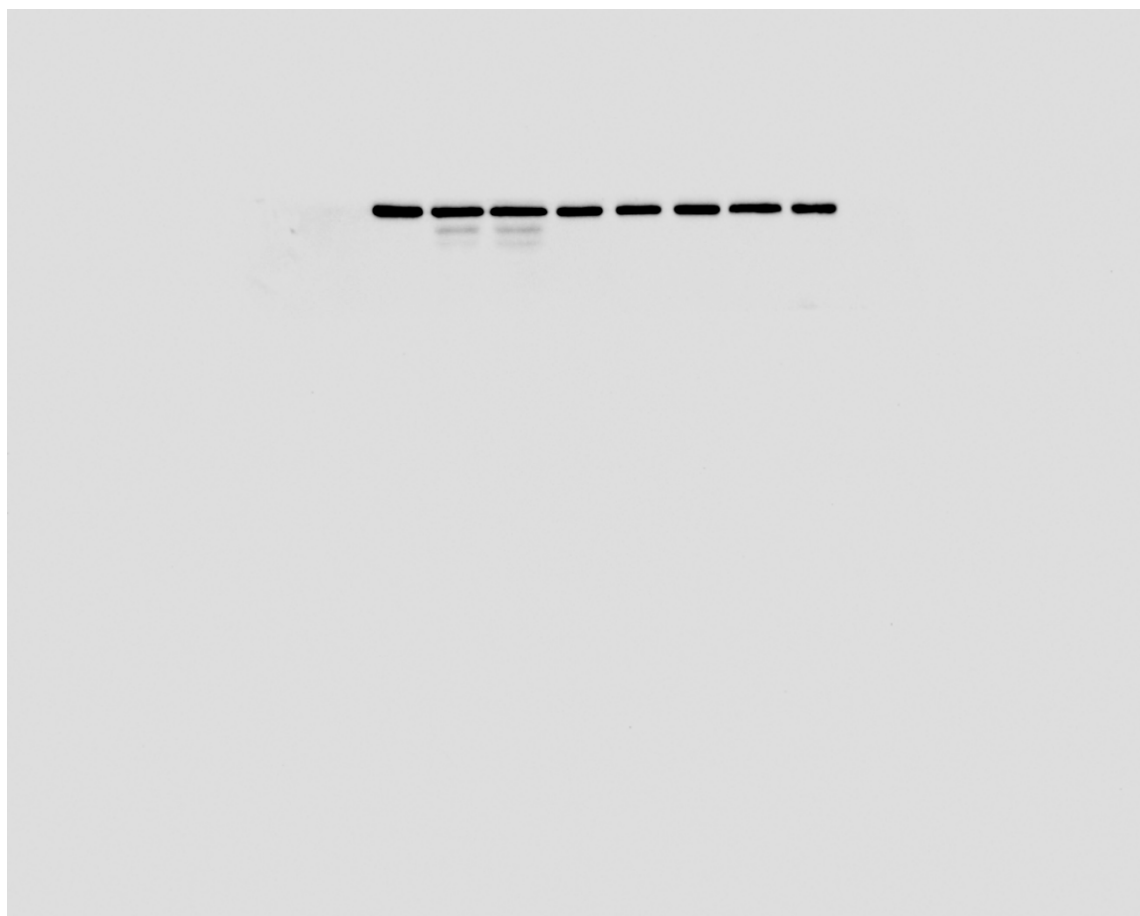

Figure. S2

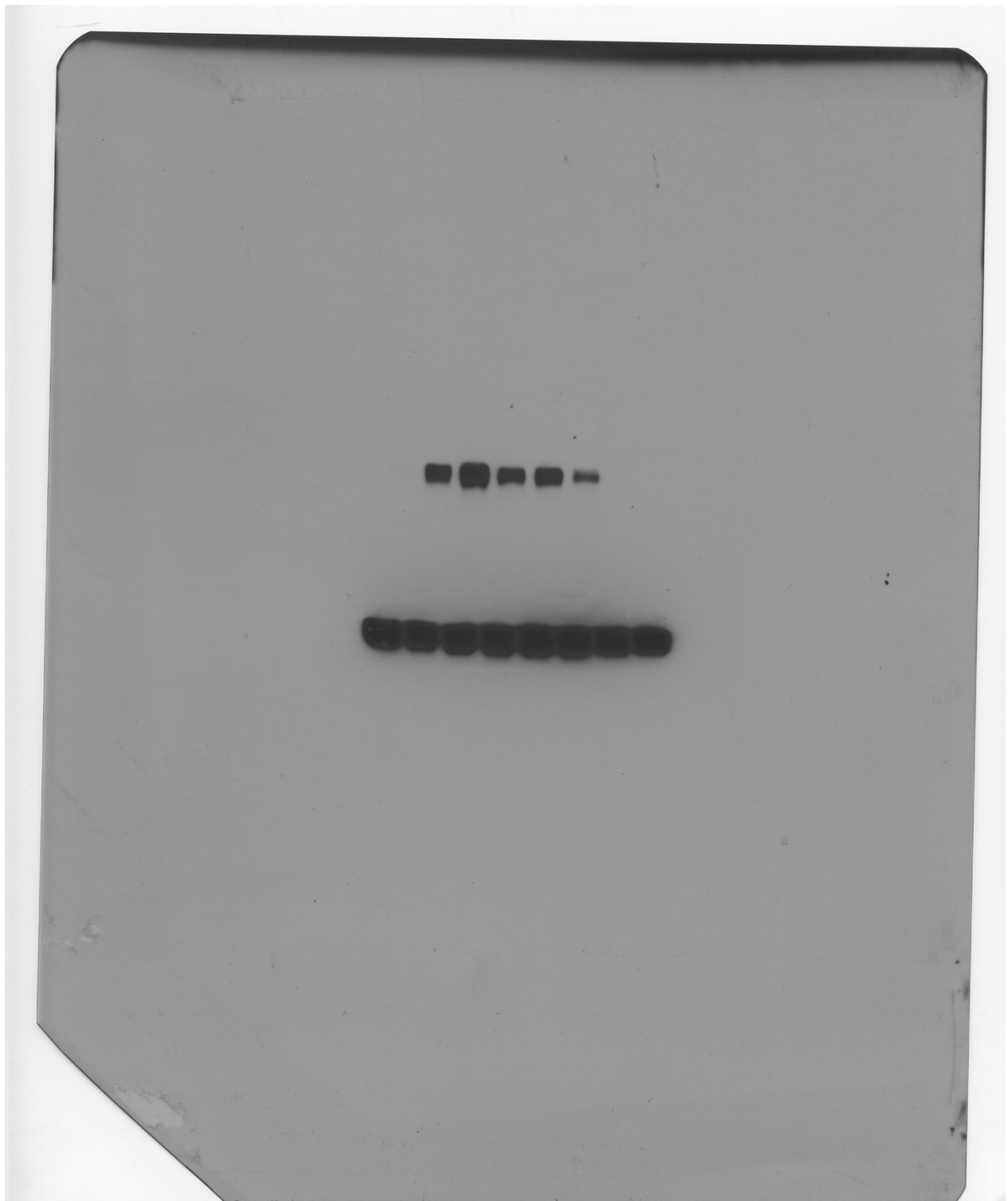

Supplement: Supplementary file 1 [file cancers-15-05844-s001.zip › cancers-2689142-supplementary/cancers-2689142-file S1.pdf]
